# The Experiences of Infertile Women on Assistant Reproductive Treatments: A Phenomenological Study

**Published:** 2012-06-30

**Authors:** B Morshed-Behbahani, L Mossalanejad, S Shahsavari, M Dastpak

**Affiliations:** 1 Department of Nursery, Jahrom University of Medical Sciences, Jahrom, Iran

**Keywords:** Infertility, ART, Phenomenology

Dear Editor,

Fertility is a significant problem in most cultures and the desire for having a child is considered as one of the human motivations in continuing life.[1] If the efforts toward becoming pregnant fail, it can cause a destructive emotional experience and is considered a severe cause of stress and disappointment with attendant risks for mental health, general well-being selfesteem and general marital relationship especially in women.[2] Following awareness of their problem, couples use assisted reproductive methods in order to fulfill their desire for having a child. Infertility treatment involves the repeated raising and dashing of hopes of a pregnancy, possibly leading to a heightened sense of distress in the face of failure to attain parenthood.[2][3] Previous infertility studies found that treatment was associated with fertility-specific distress beyond the effect of infertility alone.[4] But in these cases, few qualitative studies have been done. Doing quantitative studies in this field such as phenomenological method, which relates to deep human emotions, has made the interviewees face limitations and also talking about emotions limits them by inadequate answers for the questions and stops them from continuing. In this research, we could become more aware of the experiences and emotions of infertile women during treatment through phenomenological method and Husserl's philosophy.[5][6]

Sampling was conducted according to saturation in women’s willingness to participate in the interviews. Seventeen women who had primary infertility were chosen. The interview was done openly and semi-structured and by the researchers themselves. Reviewing and coding the interview papers were done by three researchers with the seven-step of Colaizzi method.[6]

After doing the interviews, in order to understand the participants' beliefs and know more about their experiences, all the points were read carefully and important parts (including rich contexts about infertility phenomenon and its treatments) were extracted and then, every important point was explained and definitions were noted as codes. Then, the codes were organized in groups and these groups were compared with the participants' real explanations for its validity. Then results were added to the study as a complete explanation of experiences in infertility and participants' infertility treatments and the way to achieve clear definitions with no ambiguity was reviewed and major titles with their subdivisions were specified. The achieved topics include 7 themes and 22 minor topics as: 1) Participants’ feelings toward infertility which included: depression, feeling guilty, loneliness and isolation, 2) Spouse's relationships with each other which included cooperation for success in treatments, improving relationship and in some case family quarrels, 3) Infertile couples' familial relationships and their relations with own families and" in-laws" which included hiding it and their feelings in family gettogethers, 4) Assisted reproductive treatments which included two important feelings, e.g. the treatments were nice, hopeful, peaceful or very hard, expensive and tiresome procedures, 5) Couples' sexual relationships which included the decrease of libido, lack of self-esteem and feel useless, 6) Couples' dreams and aspirations which included stressful dream, being afraid of losing a valuable blessing and achieving a position in society and family, and 7) Financial problems which included costly treatments, lack of insurance coverage and the expenses of commuting.

Analyzing the couples' statements showed that infertility had some various and deep effects on couples and their relationships. It evidently made ladies feel disappointed, worried, angry, and being useless and guilty, which resulted in that they made themselves isolated. Like as Imeson and et al. and Fahami who found four key themes emerged from their data: Life changes; powerlessness; hope-disappointment cycle; and social isolation.[7][8] Also, as Khodakarami and her colleagues found through their study, infertility could weaken or improve couples' relationships. As having a child is most couples' only reason to have sexual relations, when it doesn't happen, they do not like to continue; so it causes the relationship to become joyless gradually. So it seems to be one of the reasons why men intend to remarry after the failure in having a baby.[9] Having a child is a stressful dream for couples .They also described a cycle, alternating feelings of hope and disappointment in Imeson study.[7] The noticeable point of this study is patients' strong aspirations about effectiveness of assisted reproductive treatments. It is better to use more suitable confrontation mechanisms in these cases such as finding proper substitutes in order to aiming to benefit the life and decreasing the disappointment originating from not being succeeded in treatments to some extend by better psychological stages. As Khodakarami et al. had mentioned through their research, financial problems and not being financially supported of infertility treatments by insurance companies were the major worries of couples.

The results of this research showed that women struggling with infertility were disappointed in their lives. They had a vast collection of negative feelings simultaneously, such as anxiety, which obligated us to present them more specialized medical and psychological supports regarding their vast social and emotional problems. Also financial and insurance aids are considered as playing an important role in medical treatments in order to reduce families' vulnerability. Last but not least, the presence of midwifery, psychological and social work teams simultaneously in specialized infertility medical clinics can present these facilities to couples properly.
